# Crystallization of Metallic Glasses and Supercooled Liquids

**DOI:** 10.3390/ma17143573

**Published:** 2024-07-19

**Authors:** Dmitri V. Louzguine-Luzgin

**Affiliations:** 1 Advanced Institute for Materials Research (WPI-AIMR), Tohoku University, Sendai 980-8577, Japan; dml@wpi-aimr.tohoku.ac.jp; Tel.: +81-22-217-5957; 2 MathAM-OIL, National Institute of Advanced Industrial Science and Technology (AIST), Sendai 980-8577, Japan

**Keywords:** crystallization, metallic glasses, supercooled liquids

## Abstract

This is an overview of recent findings on the structural changes observed upon heating, including crystallization processes in conventional metallic glasses, bulk metallic glasses, and their corresponding supercooled liquids. This paper encapsulates the various crystallization behaviors in metallic glasses by primary, eutectic, and polymorphous mechanisms, highlighting the complexity and diversity of the nucleation and growth mechanisms involved. Mechanically induced room-temperature crystallization is also discussed.

## 1. Introduction

For a long time, the predominant structure type of bulk engineering metallic alloys has been crystalline. An Au-Si alloy formed an amorphous structure after rapid solidification at an exceptionally high cooling rate of 10^6^ K/s [1]. Later, Pd-Cu-Si [2] and Pd-Ni-P [3] were found to be the best metallic glass formers, produced in bulk via flux treatment to suppress heterogeneous nucleation. In the last 15 years, advancements have enabled the production of bulk metallic glasses with dimensions of at least 1 mm in every spatial dimension, sparking widespread scientific interest and leading to specialized international conferences [4,5]. Bulk metallic glasses [6,7] have a unique supercooled liquid state that inhibits crystalline nucleation, allowing for the production of samples with thicknesses of 1 to 100 mm through various casting processes [8]. They are metastable at room temperature and undergo devitrification/crystallization upon heating [8], irradiation [9,10], or intensive mechanical impact at room temperature [11], forming nanostructures and enhancing mechanical properties. This process is now a common method for producing metallic nanomaterials with superior properties, especially magnetic properties. Some special attention is paid to nearly equiatomic compositions called high-entropy metallic glasses [12,13], some of which show a glass-to-glass transition [14,15].

Most metallic glasses are crystal nucleation-controlled while some of them contain crystal nuclei, and thus, are growth controlled. For example, Pd_42.5_Cu_30_Ni_7.5_P_20_ is a true bulk glass former containing no crystalline particles or nuclei, while Pt_42.5_Cu_27_Ni_9.5_P_21_ is a crystal growth-controlled bulk glass former containing nanoparticles around 1 nm in size of a different chemical composition [16]. These structural distinctions influence their room-temperature mechanical properties: the latter alloy is significantly more ductile in compression than the former one.

Of course, there are instances when the glass-forming ability of an alloy is insufficient and a crystal–glass composite is formed, or when heterogeneous nucleation occurs on an oxide inclusion. However, when the volume fraction of crystals is low, they typically do not affect the properties unless the oxide inclusions act as stress concentrators. This is quite common in lanthanide-based alloys with a high affinity for oxygen [17].

## 2. Glass Formation from Liquids

Formation of glasses takes place by rapid enough cooling of a liquid from above its liquidus temperature (*T*_l_) to below the glass-transition temperature (*T*_g_) when it solidifies (vitrifies) without crystallization [18]. The glass transition process is a complex phenomenon [19], and its consideration is beyond the scope of this review. Many theories describe the glass transition [20,21]. It appears as a second-order phase transformation, with continuity in material volume and entropy, but discontinuity in their derivatives [22]. However, there are reasons to suggest that the glass transition could be a first-order transition [23,24].

Among various factors influencing the glass-forming ability of a liquid alloy, such as the reduced glass-transition temperature (*T*_rg_ = *T*_g_/*T*_l_), one can particularly mention its fragility: deviation from the Arrhenius law for the temperature dependence of the viscosity (*η*) [25,26]:

(1) $$\eta =\eta_{0}\exp(E_{a}/RT)$$

where *η*_0_ is a pre-exponential factor, *R* is the gas constant and *E_a_* is an activation energy for viscous flow. The viscosity of an equilibrium liquid exhibits an Arrhenius temperature dependence with a constant activation energy at a moderately high temperature above *T*_A_ (deviation from the Arrhenius law temperature), but *E_a_* increases on cooling of all supercooled liquids (both strong and fragile but to varying degrees). The origin of liquid fragility is under scientific investigation. One approach indicates that the structural changes in the supercooled liquid are responsible for liquid fragility [27]. Here, deviation of the temperature dependence of viscosity of a supercooled liquid from the Arrhenius equation takes place through modification of the activation energy by structural changes in the liquid. Other authors suggest that at a lower temperature, the activation energy increases due to cooperative dynamics among atoms as the time for local structural changes becomes comparable to the phonon lifetime, leading to super-Arrhenius behavior [28]. Here one should mention that at ultrahigh temperatures, even in supercritical liquids under supercritical pressure when boiling is suppressed, the viscosity starts to rise again [29]. The electronic structure was also reported to be responsible for liquid fragility [30]. The effects of external pressure on shear viscosity were also studied [31].

## 3. Structural Relaxation and Phase Separation on Heating Prior to Crystallization

Metallic glasses exhibit complex relaxation phenomena upon heating. Relaxation can be divided into: (a) slow, high-temperature α-relaxation, involving the entire glass and responsible for the glass–liquid transition and vice versa [32], and (b) fast, low-temperature β-relaxation [33], in localized regions, responsible for mechanical properties at room temperature. Here, relaxation, in general, can be defined as the time-dependent response to external actions like mechanical loading at a certain temperature and can be reversible on cooling and heating. Structural relaxation involves gradual structural changes (densification) toward a more stable glass on heating, starting at the beginning of relaxation temperature (*T*_br_). This is irreversible below *T*_g,_ and the sample must be heated up to the supercooled liquid to restore the excess volume on subsequent cooling.

Usually, structural relaxation [34] leads to embrittlement of the glasses. However, low-temperature annealing at temperatures close to *T*_br_ can lead to the opposite. For example, despite expectations of embrittlement, the Cu_36_Zr_48_Al_8_Ag_8_ bulk glassy samples annealed at a low temperature of 200 °C exhibited ductilization rather than embrittlement [35]. The annealing process induces structural relaxation, leading to densification and stress release, resulting in reduced hardness but increased room-temperature compressive stress and plasticity. However, high-temperature annealing near *T*_g_ led to a reduction in plasticity. Fracture surfaces exhibited dimple patterns in as-cast samples and vein patterns in annealed samples, suggesting differences in crack propagation speed and stress states. The reason for ductilization could have been phase separation, but the atomic-scale elemental mapping showed no phase separation at this temperature [35]. Phase separation in this alloy takes place at a higher temperature. Structural relaxation is mostly a topological process leading to the densification of the glassy phase, while phase separation is related to the changes in local chemistry.

Spinodal phase separation into two glassy phases was observed in the Zr-Y-Al-Ni [36] system and Zr-La-Al-Ni-Cu [37] system alloys. Phase-separated areas crystallize at different temperatures. For example, a residual globular glassy phase in the Cu_35_Zr_45_Ag_20_ alloy annealed at 722 K for 1 ks retained its glassy structure while the residual glassy matrix crystallized [38]. Phase separation was also observed in Zr-Cu-Fe-Al alloys on heating or deformation [39] and prevented embrittlement of the glassy samples after relaxation [40]. When the binodal phase separation mechanism is activated, then the separated particles precipitate as far from each other as possible in the glassy matrix and enable tensile ductility in the Zr-Cu-Fe-Al BMG after thermomechanical treatment [41]. In these alloys, phase separation occurred owing to repulsive interaction between Zr-Y, Zr-La, Cu-Ag, and Cu-Fe atomic pairs. Phase separation was also found in a Zr_52.5_Ti_5_Cu_17.9_Ni_14.6_Al_10_ glassy alloy without immiscible elements [42].

## 4. Crystal Nucleation and Initial Crystallization Stage

The glasses, once formed, retain their structure without crystallization for a long time. Recent results on metallic glasses stored at room temperature for at least 15 years showed that most of them retained their initial structure with only a moderate decrease in crystallization temperature (unless severely oxidized), confirming their high stability at ambient conditions [43]. The alloys with low *T*_g_ like Ce-based ones were reported to increase their stability in long-term aging [44]. However, all metallic glasses crystallize on heating.

The mystery surrounding crystal nucleation in liquids and glasses stems from several complex factors. Crystal nucleation occurs on a tiny scale and at rapid timescales, making it challenging to directly observe or capture in real-time experiments that limit our understanding of the initial stages of nucleation. Here computer simulation is a procedure that can help. Modeling and simulating nucleation processes computationally involves complexities due to the numerous atoms involved and the need to capture both short- and long-range atomic interactions accurately [45,46]. Moreover, the crystal nucleation process can be heterogeneous and homogeneous. Balancing thermodynamic barriers and kinetic stability during nucleation involves complex interactions. Due to these complexities and limitations in observation and modeling, the precise mechanisms and pathways involved in crystal nucleation in liquids and glasses remain a fascinating yet challenging area of study [47]. Thus, understanding how nucleation initiates and progresses within the context of energy landscapes and energy barriers is still an ongoing area of research [48,49].

**Figure 1:**
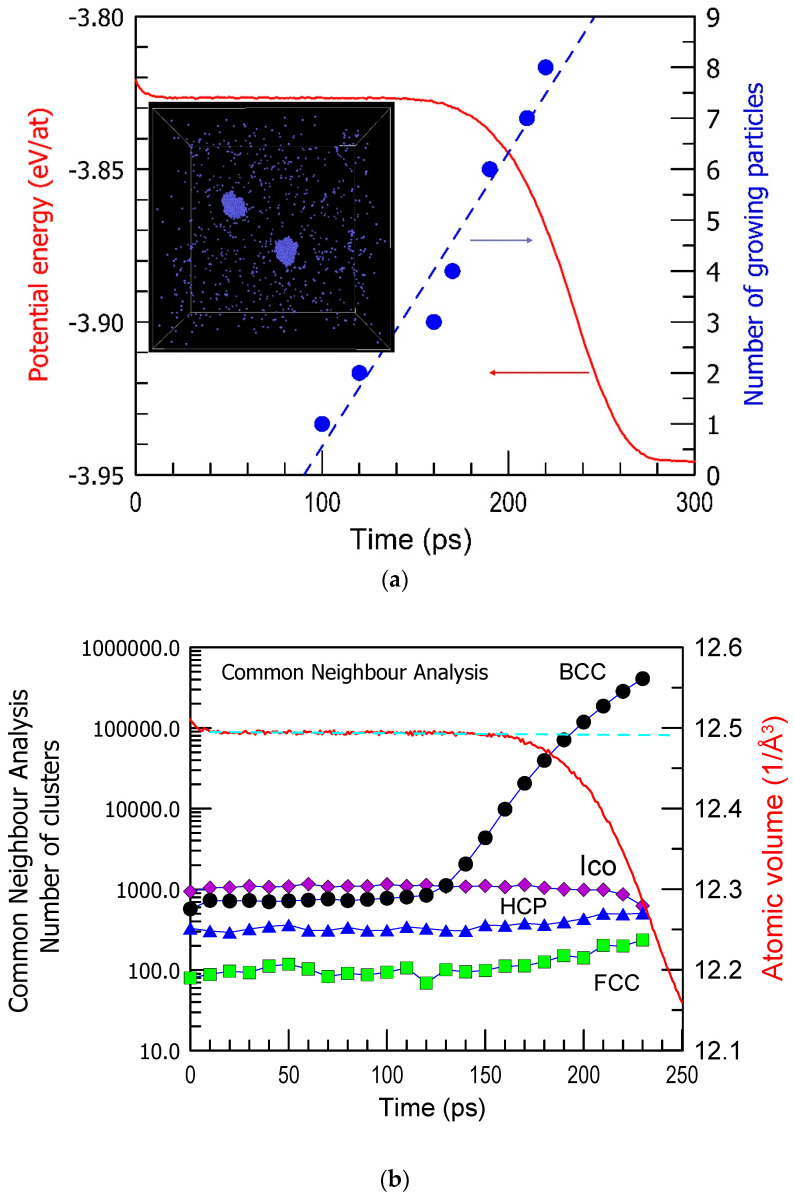
(**a**) The number of growing BCC Fe particles and the potential energy (PE) per atom as a function of simulation time at 1100 K in a simulated cell shown in the inset (a stage at 150 ps when 2 BCC crystals are growing). The arrows indicate the corresponding axes. (**b**) The number of atoms belonging to FCC, BCC, HCP, and Icosahedral (Ico) clusters result from common neighbor analysis as a function of simulation time. The results are obtained from a series of computations in Ref. [50].

The incubation period in crystal nucleation is still an intriguing phenomenon. It refers to the time between the onset of conditions favorable for nucleation and the actual appearance of the first crystal nucleus (Figure 1a) [50]. This period is associated with a lack of observable changes, making it particularly mysterious. During the incubation period, there might be subtle, undetectable changes occurring at the atomic level, such as the formation of small clusters or structural rearrangements, which are challenging to observe directly (for example, BCC Fe in Figure 1b). The incubation period is not the only mysterious aspect of crystal nucleation, it holds significant importance in understanding the mechanisms of nucleation. Why does the system wait for a certain time with no visible changes in structure and then undergo nucleation and growth of many crystalline nuclei like in Figure 1b? What is a trigger of nucleation in various regions? Unraveling its nature remains a challenging yet essential research task in materials science and physics. It is also important for predicting the glass-forming ability of alloys, and the role of crystal–glass interfaces is emphasized [51,52]. The crystal–glass interfaces play an important role in improving the mechanical properties of metallic glasses like in situ formed composites.

Crystallization of pure Cu [53,54], Ni [55], and other metals [56] was studied substantially by computer simulation and compared with the experiment [57]. The energy barriers for the crystallization of pure metals were also determined [58]. In a cell of Fe, kept at 1100 K (see Figure 1a inset), nucleation happened after the incubation period of 100 ps, and the first nucleus is observed well before the changes in the potential energy become visible. Moreover, no clear changes in the structure of a liquid are observed within the incubation period. Equilibration of a liquid at 1100 K cooled at 10^13^ K/s from 2500 K took about 20 ps when the potential energy of the system became stable. Yet, no structural changes in terms of the atomic clusters are seen for the next 80 ps (Figure 1b). Recent molecular dynamics studies also suggested that contrary to classical nucleation theories, liquid atoms join crystal clusters by changing their local order parameter with minimal movement and do so cooperatively rather than individually [28].

Modelling of Cu-Zr alloys showed that small additions of Zr (1–10 at.%) to Cu significantly increase incubation time and slow down crystal growth, enhancing glass formation. Detailed molecular dynamics simulations reveal that the critical cooling rate for glass formation increases substantially with Zr content, and despite low equilibrium solubility, growing FCC Cu crystals can dissolve a few atomic percent of Zr, exhibiting a concentration gradient at higher Zr contents and polymorphic growth in supersaturated alloys [59].

**Figure 2:**
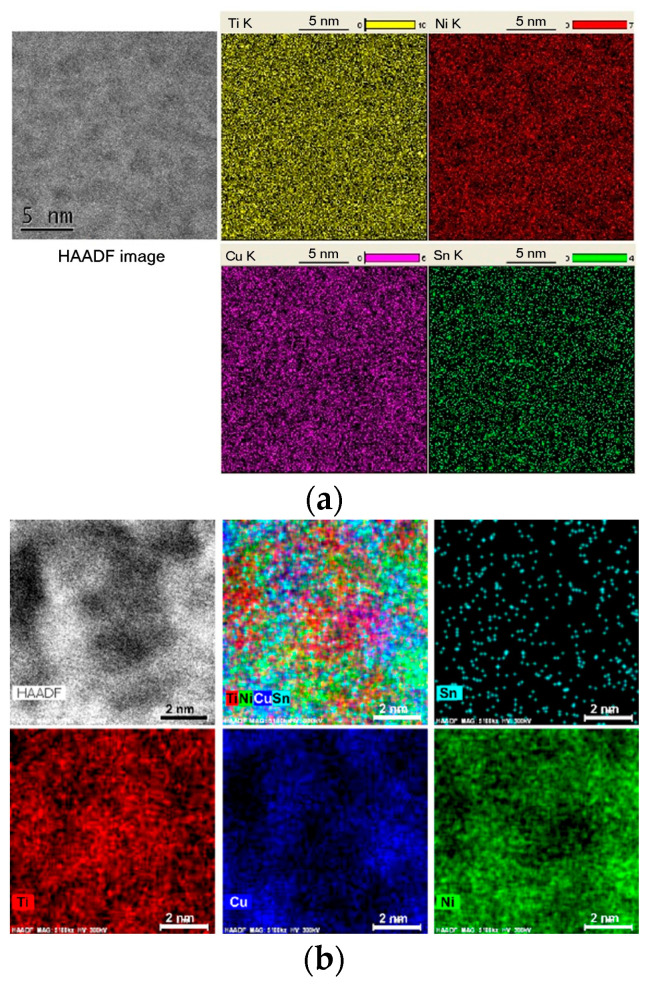
High-angle annular dark-field and scanning transmission electron microscopy energy-dispersive X-ray elemental mapping (as indicated) of the as-cast Ti_50_Ni_23_Cu_22_Sn_5_ glassy alloy sample (**a**) and the sample annealed at 753 K for 40 s as indicated (**b**). Reproduced from [60] with permission of Elsevier.

The existence of an incubation period in multicomponent metallic glasses can be explained by the time required for chemical ordering in the matrix liquid or glassy phase. The Ti_50_Ni_23_Cu_22_Sn_5_ bulk glass-forming alloy, initially homogeneously glassy, undergoes nucleation preceded by the chemical ordering of a liquid [60]. Nanoscale Ti-enriched zones and regions enriched in Ni and Cu form during the incubation period, becoming chemically ordered while remaining structurally disordered (Figure 2). This chemical ordering reduces the nucleation energy barrier for the cF96 crystalline phase, which begins to precipitate after the incubation period. The process resembles the formation of Cu-rich Guinier–Preston zones in Al-based alloys, preceding intermetallic phase formation.

## 5. Growth of Crystals and Kinetics of Crystallization

Crystal growth follows nucleation. The growth velocities in metallic glasses range from very low values (down to 10^−1^ nm/s) in the case of primary crystallization [61], up to 10^2^ m/s in the case of pure metals [62] and chalcogenide glasses [63]. Eutectic colonies exhibit intermediate growth rate values (~100 nm/s) [64]. Fast surface-induced crystallization also takes place in some glasses [65]. A special group of metallic glassy alloys undergoes peritectic-like transformations with the participation of nanocrystalline and nanoquasicrystalline phases. This process involves the dissolution of one phase in an amorphous matrix and the release of another phase during a one-step transformation [66].

**Figure 3:**
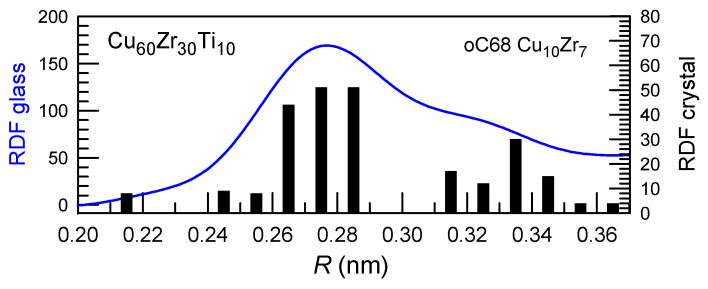
Radial distribution functions (RDF) of the Cu-Zr-Ti glassy alloy in the first coordination shell (blue). Black bars show the corresponding radial distribution functions of the Cu_10_Zr_7_ intermetallic compound integrated at 0.01 nm step. Good correction is observed indicating similarities in local structures leading to a reduced energy barrier for nucleation. Reproduced from [72] with the permission of Elsevier.

Kinetics of the crystallization processes in metallic glasses is analyzed by Kolmogorov [67]—Johnson–Mehl [68]—Avrami [69] general exponential equation for the fraction transformed *x*:

*x* = 1 − exp(−(π/3)·*Iu*^3^*t*^n^) (2)

where *I* is the nucleation rate, *u* is the growth rate, and *t* is time. The exponent *n* value of about 4 corresponds to nucleation and interface-controlled growth while those close to 2.5 correspond to nucleation and diffusion-controlled growth. Usually, this law is well maintained, but one should mention that in some cases exceptionally high n exponent values are found in Cu-Zr-Al alloys like Cu_50_Zr_45_Al_5_ [70] and Zr_45_Cu_49_Al_6_/Zr_46_Cu_46_Al_8_ [71] with high values of n exponent in which the nucleation rate of the Cu_10_Zr_7_ phase with complex oC68 crystalline structure increases drastically with time. This crystal structure is quite close to that of some Cu-Zr-based metallic glasses (Figure 3) [72], and thus, easy nucleation with a low energy barrier could be expected.

The time–temperature-transformation diagrams created in the isothermal mode or under continuous heating are useful for comparison of the thermal stabilities of different glasses [73,74,75]. A comparison of the long-term thermal stabilities of different metallic glasses was performed using continuous heating transformation diagrams [76] constructed by applying a corollary from the Kissinger analysis method using the DSC data at different heating rates including ultrafast DSC [77]. Such diagrams can be recalculated from the isothermal diagrams by analogy with those for phase transformations in steels [78].

Metallic nanoglasses [79] and stabilized (ultrastable) glasses [80] have distinct crystallization mechanisms. The differences in the crystallization pathways between as-case and selectively laser-melted (SLM) metallic glasses are attributed to differences in the oxygen content. High oxygen content (~1 at%) in SLM samples reduced the thermal stability of the Zr-based BMG.

## 6. Fast Primary Crystallization

Nanocrystals form with higher nucleation rates and lower growth rates compared to microscale crystals, in which the nucleation rate is generally lower, and the growth rate is higher. The formation of nanocrystals often requires large undercooling. Nanocrystallization causes a high density of grains and grain boundaries due to the small size of the grains. Nanocrystallization usually occurs when the alloy is far from equilibrium, with rapid nucleation at limited atomic mobility. Once formed nanostructures are often metastable and undergo phase transformation upon heating with final transition towards microcrystalline structures. Macrocrystalline structures tend to be more stable under similar conditions.

Nanocrystals are usually formed upon primary crystallization. Glassy and partly nanocrystalline Fe-based alloys with fine Fe nanoparticles [81,82] are of high industrial importance owing to their excellent soft magnetic properties, especially at high frequencies. Their properties are constantly being improved [83]. A high crystal nucleation rate was observed in high-entropy type (Fe_0.25_Co_0.25_Ni_0.25_Cr_0.125_Mo_0.125_)_100-x_B_x_ alloys [84].

Al-based metallic glasses exhibit the formation of primary nanoscale Al phase by diffusion-controlled growth [85,86]. They are found to contain spatial heterogeneities consisting of Al-rich regions. They contribute to an increase in the density of Al nanocrystals [87,88]. A nano-dispersed structure was obtained directly from the melt upon rapid solidification of the Al-Y-Ni-Co-Pd alloys [89]. The addition of a high amount of Y increases *T*_g_ [90]. An extremely high density of precipitates in the order of 10^24^ m^−3^ was obtained.

**Figure 4:**
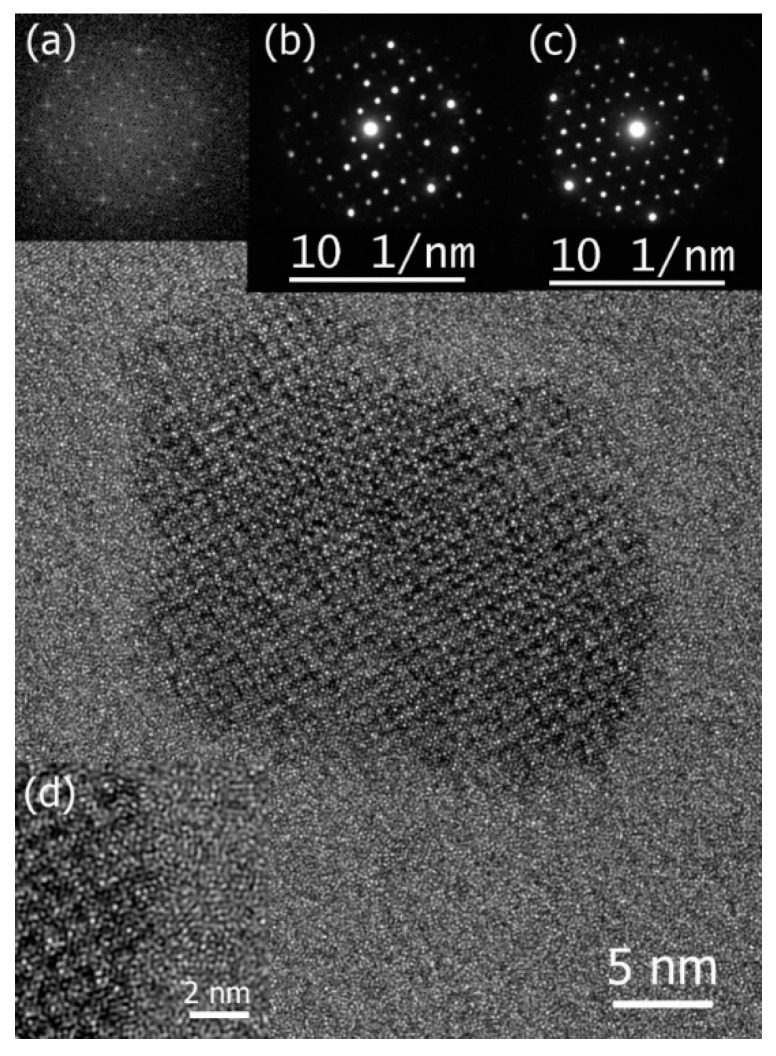
High-resolution TEM image of an Al_19_Fe_4_MnSi_2_ nanoparticle and the insets: its fast Fourier transform (**a**) and two NBD patterns (**b**,**c**) were indexed according to alpha phase lattice with zone axes of [100], [100], and [111], respectively. In both cases, the reflections closest to (000) in distance are of {110} type. Strong reflections at the edge of the patterns in (**b**,**c**) are of {611} type. (**d**) a close-up view of the interface. Reproduced from [91] with permission of Elsevier.

A thorough examination of the primary crystallization process within an Al-Fe-Mn-Si metallic glass [91] showed that primary crystallization occurred via homogeneous nucleation. The crystal growth rate of the alpha Al_19_Fe_4_MnSi_2_ phase formed in the Al_68_Fe_10_Mn_4_Si_18_ glassy alloy (Figure 4) was directly measured in TEM, allowing the estimation of the diffusion coefficient, which was compared with existing literature data. Remarkably, the diffusion of Mn, the limiting element, exhibits atomic mobility approximately three orders of magnitude faster than that in Al crystal and in the AlFe intermetallic compound. While the relatively high diffusion coefficient suggests unrealistically low viscosity, challenging the applicability of the Stokes–Einstein relationship, the absence of an endothermic signal associated with glass transition below *T*_x_, even with Fast Differential Scanning Calorimetry (FDSC), alongside a high *T*_rx_ value, suggests that Al_68_Fe_10_Mn_4_Si_18_ could be a strong glass-former that does not exhibit a glass transition upon heating below *T*_x_. Furthermore, the significantly accelerated growth of the crystalline phase compared to estimates derived from diffusion coefficients for pure Al and the AlFe compound hints at a possible collision-limited growth regime. This accelerated growth may be facilitated by thermodynamically enhanced atomic mobility at the crystal/glass interface due to chemical potential effects.

The addition of rare-earth metals was found to cause nanocrystallization of Mg-Ni metallic glasses [92]. A unique primary crystallization has been observed recently in the Mg_75_Ni_20_Mm_5_ metallic glass [93]. The analysis revealed that despite significant solute partitioning and soft impingement of diffusion fields around neighboring crystals, the growth remains linear, suggesting interface-controlled kinetics. The crystals exhibited a constant aspect ratio during growth, defying dendritic interfacial instability typically associated with solute partitioning. Remarkably, the primary Mg_2_Ni crystals grow with a composition close to Mg_9_Ni_9_RE, indicating supersaturation with Ni because of partially vacant Mg sites. The constant isothermal growth rate and absence of dendritic growth are attributed to the wide range of atomic diffusivities below the glass-transition temperature. Specifically, Ni, the fastest diffusing species, preferentially partitions into the growing Mg_2_Ni crystals, while the immobile RE atoms remain uniformly distributed.

Very small nanoscale quasicrystals of about 10 nm in size and even below with a very high density of precipitates were formed on devitrification of the Zr-Al-Ni-Cu-Ag [94], Hf-Pd-Ni-Al [95], Hf-Al-Ni-Cu-Pd [96], and Cu-Zr-Ti-(Pd,Au) [61] alloys owing to easy nucleation of the icosahedral phase having low interfacial energy with the glassy phase [97].

## 7. Truly Eutectic Crystallization and Crystallization Looking like Eutectic

**Figure 5:**
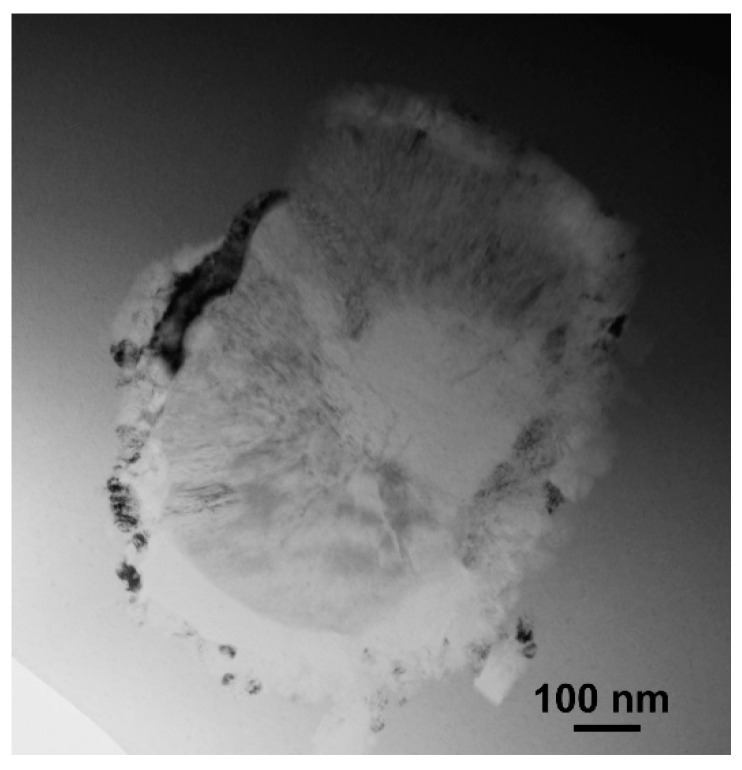
Bright-field image of the eutectic colony in the annealed Zr_62.5_Cu_22.5_Al_10_Fe_5_ glassy sample, TEM.

Many metallic glasses crystallize by the classical eutectic reaction when several crystalline phases crystallize simultaneously forming eutectic colonies as was found in Pd-Ni-P-Si (spherical colonies) [64] and Zr-Cu-Fe-Al alloys (rod-type colonies) [98] (Figure 5), for example.

**Figure 6:**
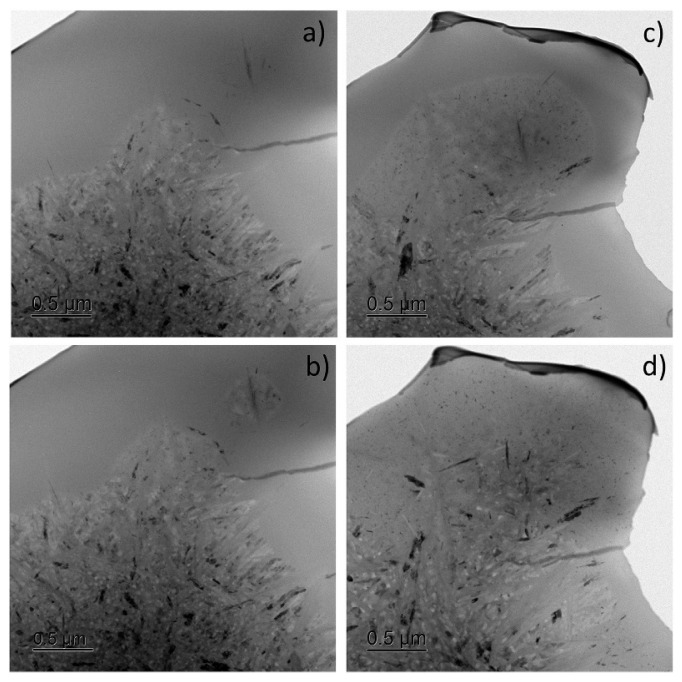
Bright-field TEM images indicating growth of a single colony towards the residual liquid phase observed in Al_85_Y_8_Ni_5_Co_2_ at: (**a**) 603 K, (**b**) 608 K, (**c**) 613 K, and (**d**) 633 K. Reproduced from [99] with permission of Elsevier.

At the same time, the detailed study of the eutectic-like reaction observed below *T*_g_ in Al_85_Y_8_Ni_5_Co_2_ (for which the phase transformation kinetics looks like the eutectic one) revealed that it is not a straightforward eutectic reaction but involves two stages: the precipitation of a primary intermetallic compound followed immediately by the crystallization of nanoscale Al around it (Figure 6) [99]. Alongside the growth of existing colonies triggered by the intermetallic compound, new needle-like intermetallic compound particles form in the glassy matrix, initiating the development and growth of new eutectic colonies (Figure 6a,b). These colonies are abnormal, often lacking boundaries between the aluminum nanoparticles and the intermetallic compound, which are separated by the glassy matrix. Despite this, the transformation statistically behaves as a single-step process with a nearly constant Avrami exponent. At higher heating rates, when the temperature quickly surpasses *T*_g_, the crystallization process shifts to the primary crystallization of a nanoscale solid solution of Al, followed by the formation of intermetallic compounds through subsequent reactions occurring at a longer annealing time or at higher temperatures.

The crystallization of a Cu_58_Y_37_Sc_5_ glassy alloy also looks like the eutectic one at first glance [100]. However, nucleation and growth of micron-scale oI12 Cu_2_Y, cP2 CuY, and cP2 CuSc phases occur heterogeneously after a defined incubation period. The crystallization kinetics suggest primary crystallization with diffusion-controlled growth, rather than eutectic crystallization, highlighting the complex nature of the process. The formation of micron-scale Y-/Sc-rich particles in the as-cast state suggests a metastable phase diagram, possibly inhibiting intermetallic compound formation. Complex crystallization was observed in an Au-based bulk metallic glass [101].

## 8. Fast Polymorphic Crystallization

Observation of polymorphic crystallization is a rare case in metallic glasses as their composition is usually closer to eutectics [102]. Recently, reactions forming cP2/B2 phase were studied in the Ti_50_Ni_22_Cu_22_TM_6_ glassy alloys [103] close to the glass-transition temperature. XRD and SAED patterns confirm the presence of cP2/B2 TiNi solid solution phase, with minor intermetallic compounds detected in Cr- and Mn-containing alloys. The Ti_50_Ni_22_Cu_22_Mn_6_ alloy showed a high *n* exponent, indicating an increasing nucleation rate with time. The crystal growth rate in the Ti_50_Ni_22_Cu_22_Fe_6_ metallic glass obtained by in situ transmission electron microscopy is orders of magnitude higher than that allowed by thermal long-range diffusion estimated from viscosity. It is suggested that the atomic transfer at the atomic-scale thick glass/crystal interface may be accelerated by the crystal/glass difference in the corresponding thermodynamic and chemical potentials [104].

## 9. Features of Crystallization during Welding and Additive Manufacturing

Welding and joining of bulk metallic glasses are performed by friction [105], laser [106], or electron welding [107]. Additive manufacturing (AM) is performed by the sintering of powders and often leads to partial crystallization of the glassy powder [108]. For a specialized review dedicated to this topic see Ref. [109]. The ability to produce metallic glass without crystallization during selective laser melting hinges on the interaction between the laser beam and the glassy material [110]. One of the important issues here is the heat-affected zone with a partly crystalline structure formed when a subsequently applied layer anneals the underlayer. AM process is influenced by powder size and shape, laser beam power, scanning speed, and other processing parameters. A Zr_52.5_Cu_17.9_Ni_14.6_Al_10_Ti_5_ bulk metallic glass fabricated AM showed yield and fracture stresses slightly lower than for its as-cast counterpart. The stress drops during serrated flow were much lower and more uniform [111]. Advancements in laser technology necessitate a deeper understanding of these interactions to enhance AM processes. Systematic studies tailored to specific AM techniques and metallic glassy systems are required, along with an understanding of crystallization kinetics during laser irradiation.

## 10. Mechanically Induced Crystallization at Room Temperature

Mechanically induced crystallization of metallic glasses has been studied substantially and reviewed recently [112]. It is known and recently proven again that mechanical alloying, using a high-energy ball milling technique, can cause both amorphization and crystallization [113]. High-pressure torsion and multiple rolling also caused structural changes and crystallization in the metallic glassy phase [114,115]. However, possible temperature rise cannot be neglected at such an intensive deformation. Here, the emphasis is given to recent findings on room-temperature crystallization.

Quite often thermal and mechanical crystallization processes lead to different crystal structures. Heating Fe_48_Cr_15_Mo_14_C_15_B_6_Y_2_ [116] and Fe_48_Cr_15_Mo_14_C_15_B_6_Tm_2_ glassy alloys formed nanoscale particles of the χ-Fe_36_Cr_12_Mo_10_ phase on crystallization [117,118] at an early crystallization stage [119] and in a Fe_50_Cr_15_Mo_14_C_15_B_6_ alloy after partial crystallization [120]. This phase was also found in Cr- and Mo-free glassy alloys [121]. Crystallization of similar Co-based alloys was also studied [122].

**Figure 7:**
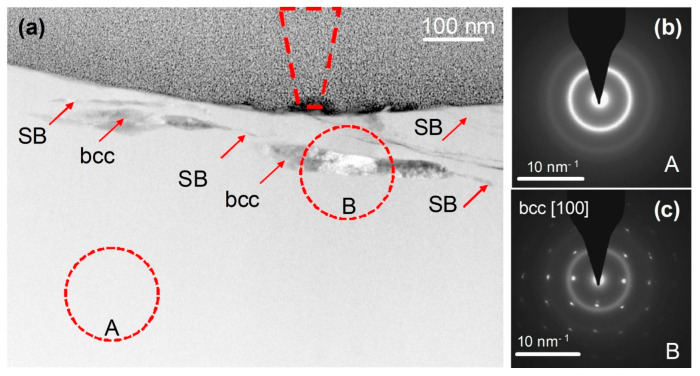
Cross-sectional TEM observation of a wear track. (**a**) HAADF STEM image of the cross-section of the track, showing the MG matrix and within it shear bands (SBs) and bcc crystals. The position and size of the AFM tip are indicated by the dashed cone located at the center of the track, which overall is 650–750 nm wide. (**b**,**c**) are SAED patterns from regions A and B, respectively, indicating a glassy phase and a crystal induced by mechanical deformation. The A and B circles on the image show the size of the selected-area aperture used for SAED acquisition. Reproduced from [123] with permission of Springer Nature.

**Figure 8:**
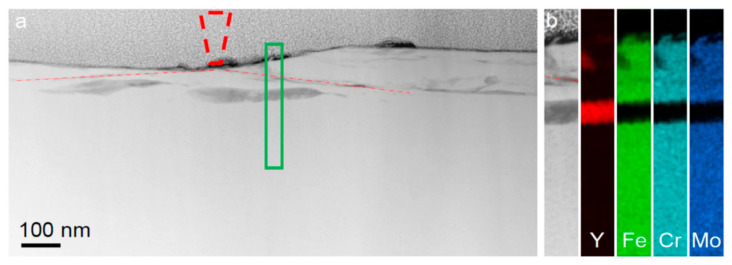
Compositional changes in the Fe_48_Cr_15_Mo_14_C_15_B_6_Y_2_ bulk metallic glass underneath the wear track. (**a**) HAADF STEM image of the cross-section of the wear track. The position of the AFM tip is shown by the dashed cone. The red dashed–dotted lines indicate the position of the shear bands generated during the wear test. (**b**) HAADF image and EDX elemental mapping of the region marked by the green rectangle in (**a**). Reproduced from [123] with permission of Springer Nature.

On the other hand, room-temperature wear of a Fe_48_Cr_15_Mo_14_C_15_B_6_Y_2_ bulk metallic glass, induced by a nanoscale diamond tip caused the formation of nanoscale layers of a bcc Y-based solid solution (Figure 7) with a composition of Y_84_Fe_8_Mo_5_Cr_3_ (at.%) directly beneath the wear track, at a depth of about 50 nm (Figure 8) [123]. The partly dark contrast of this crystalline phase in the HAADF image can relate to the fact that Y^39^ (molar volume: 19.88 cm^3^) having a significantly larger atomic number z = 39 than Fe^26^ (molar volume: 7.09 cm^3^) also has a much larger atomic size and thus almost three times higher atomic/molar volume. Thus, the dark contrast might have arisen from the three times lower atomic number density (*ND*) of Y *ND* = 0.030 Å^−3^ compared to that of Fe (*ND* = 0.085 Å^−3^).

This notable concentration of yttrium, characterized by its low thermal diffusivity in Fe, is attributed to a mechano-chemical pumping action in the heavily sheared regions [124] under the diamond tip. In bulk metallic glasses, larger atoms like yttrium move toward the central plane of the shear band (with high excess volume), consistent with findings in colloids and granular media. The extreme shear rate gradients in metallic glass shear bands facilitate yttrium concentration on shear planes which is enhanced by the soft Fe-Y interatomic potential increasing yttrium mobility. This mechano-chemical pumping effect is of fundamental scientific interest and suggests potential applications. Computer simulation also indicated low-temperature, stress-driven crystallization [125].

Room-temperature cyclic mechanical loading in the elastic mode also can induce structural changes in the glassy phase, altering its mechanical and thermal relaxation behavior on heating [126]. The thermal and mechanical relaxation spectra of a Zr_64_Cu_21_Fe_5_Al_10_ bulk metallic glass were examined before and after cyclic loading up to 0.4% elastic strain. This loading caused partial mechanically induced nanocrystallization after 10,000 elastic loading cycles at room temperature, affecting the relaxation behavior on subsequent heating experiments [127]. The nanocrystals being metastable dissolve in the glassy matrix on subsequent thermal relaxation. Deformation-induced crystallization reactions during bending were observed in a Pd-Cu-Ni-P metallic glass on the compressed side [128]. The changes in the crystallization kinetics under external loading have also been studied recently [129,130].

## 11. Summary

Crystal nucleation in liquids and glasses is a complex phenomenon. Direct observation is challenging, but computer simulations help to model these processes, revealing realistic atomic interactions. Despite some research advances, the precise mechanisms of nucleation and the role of chemical ordering in multicomponent systems continue to be a vital research area. Crystallization in metallic glasses involves diverse crystal growth velocities, from very slow primary crystallization (including nanoscale quasicrystals) to rapid polymorphic processes. Recent findings indicate that primary crystallization involves diffusion-controlled growth, while Mg-Ni-Mm glasses show interface-controlled kinetics. Eutectic crystallization forms colonies in some alloys, whereas eutectic-like reactions can involve complex multi-stage processes, as seen in Al-Y-Ni-Co alloys. Transformation kinetics are usually analyzed by the Kolmogorov–Johnson–Mehl–Avrami equation. Time–temperature–transformation diagrams are built to compare the thermal stabilities of metallic glasses. Mechanically induced crystallization occurred at room temperature, as evidenced by nanoscale particle formation in Fe-Cr-Mo-C-B-Y and Pd-Cu-Ni-P alloys and structural changes due to cyclic loading in Zr-Cu-Fe-Al glasses, demonstrating the complex nature of crystallization in metallic glasses. Crystallization mechanisms in metallic glasses need continued research to understand these processes and their implications on material properties.

## Data Availability

The data will be available on request.
